# Supplementary material for: Neuronal activity under transcranial radio-frequency stimulation in metal-free rodent brains in-vivo
Source: Commun Eng. Author manuscript; Available in PMC 2023 Dec 20. (PMC10732550; doi:10.1038/s44172-022-00014-7)
Supplement: Neuronal activity under transcranial radio-frequency stimulation in metal-free rodent brains in-vivo [file NIHMS1899100-supplement-Neuronal_activity_under_transcranial_radio-frequency_stimulation_in_metal-free_rodent_brains_in-vivo.pdf]

Supplementary Information

Neuronal activity under transcranial radio-frequency stimulation in metal-free rodent brains in-vivo

Omid Yaghmazadeh[1,*,#], Mihály Vöröslakos[1,#], Leeor Alon[3], Giuseppe Carluccio[3], Christopher Collins[3], Daniel K. Sodickson[1,3] and György Buzsáki[1,2,*]

[1]Neuroscience Institute, [2]Department of Neurology, and [3]Department of Radiology, School of Medicine, New York University, New York, NY 10016, USA

[#] These authors contributed equally
* Correspondence: Gyorgy.Buzsaki@nyumc.org or Omid.Yaghmazadeh@gmail.com

Supplementary Figures

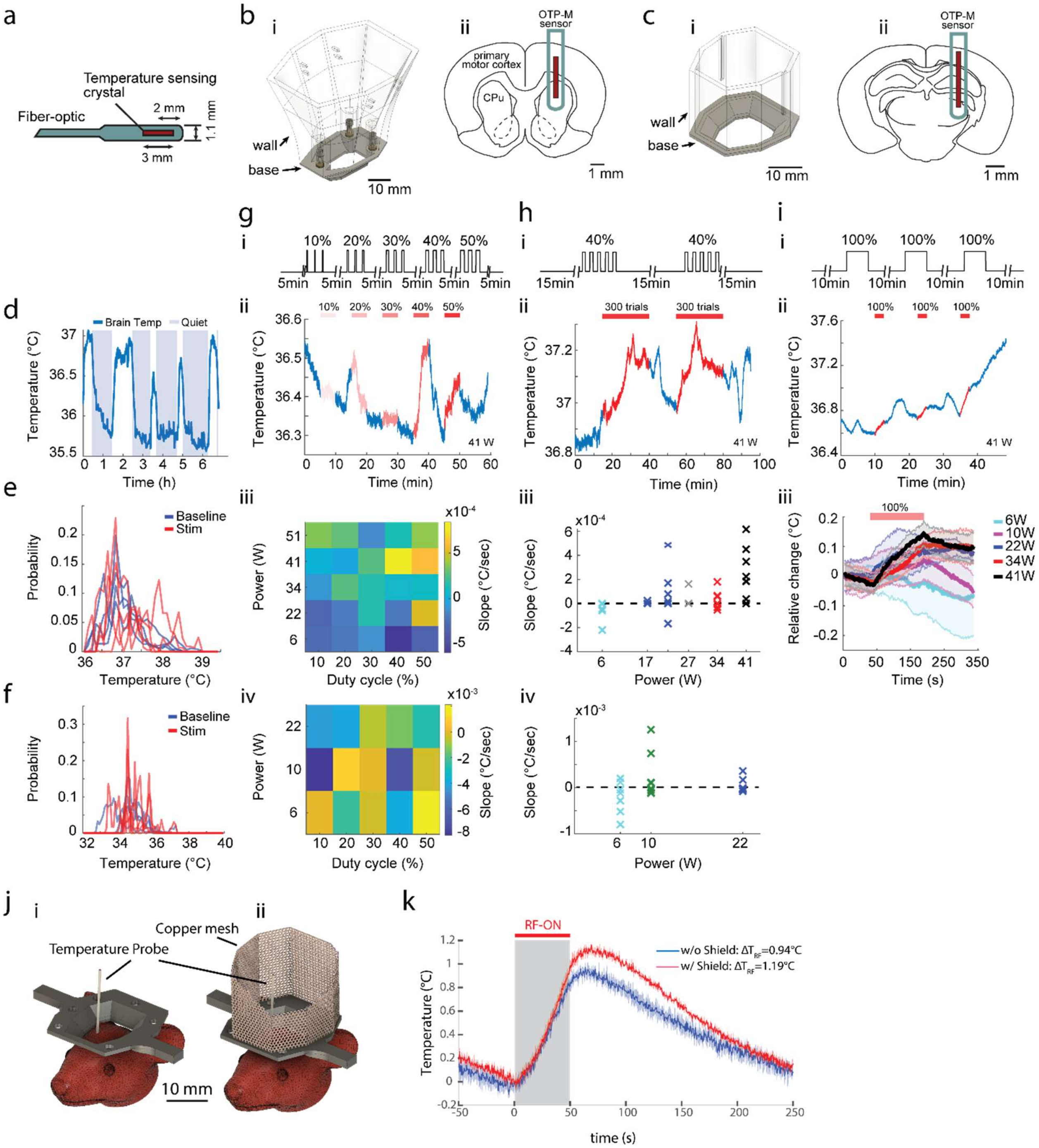

Supplementary Figure 1. **RF-induced temperature changes in electrophysiological experiments in rodents in vivo. a)** Optical temperature sensor used in vivo. **b)** i: Custom-designed plastic cap for rats (9.8 g). Base is attached to the skull with dental cement. Wall protects the optic sensor mechanically. ii: Temperature sensor is implanted into putamen (CPu) in rats (AP 2 mm and ML 2 mm). **c)** i: Custom-designed plastic cap for mice (1.7 g). ii: Optic sensor is implanted into hippocampus in mice (AP -2 mm and ML 2 mm). Note that the optic sensor is in scale. **d)** Time course of physiological temperature variation in a chronically implanted mouse. Increased temperature epochs correspond to waking/movement based on

video recording of the animal behavior. **e**,**f)** Distribution of brain temperature of rats (e) and mice (f) in each animal during baseline recordings and RF exposure (n=4 rats and 4 mice). Note that RF-induced thermal effects remain within the physiological brain temperature range. **g** to **i**) RF-induced temperature change (°C/s) in the prefrontal cortex of freely moving rats or in the hippocampus of freely moving mice. **g)** i: Schematic of stimulation paradigm (n = 60 trials at 6, 17, 27, 34 and 41 W 5s pulses, duty cycles of 10, 20, 30, 40 and 50 %). ii: Single session example (red bars denote different duty cycles at 41 W). iii,iv**:** **I**ncreasing power and/or duty cycles lead to higher brain temperature changes during stimulation both in rats (3) and mice (4). **h)** RF exposure used in our electrophysiology experiments does not induce considerable heating in brain tissue. i: Schematic of stimulation paradigm (5s - 40% duty cycle pulses, n=300 trials). ii: Single session example (red bars denote RF-ON period at 41 W). iii,iv: Each dot represents the change in brain temperature during RF exposure in rats (3) and mice (4). **i)** Continuous (100% duty cycle) RF-induced temperature effects at various RF power levels. i: Schematic of stimulation paradigm (150s - 100% duty cycle pulses, n = 3 trials). ii: Single session example (red bars denote the RF-ON period at 41 W). iii: **I**ncreasing power levels (6, 10, 22,34 and 41 W) lead to increasing brain temperature changes (mean ± SEM are shown for each power level). **j,k)** Shielding for electrophysiological recordings does not reduce RF absorption in the brain. **j)** 3D schematic of the experimental set-up (excluding the antenna and head-fixation stand which is the same as in Fig. 1d). A thin (300µm diameter) optical temperature probe (Osensa, Canada) is implanted in the hippocampus of urethane anesthetized mouse and fixed to the skull; without (i) and with (ii) adding the copper mesh shielding used in the chronic electrophysiological experiments. **k)** Comparison of the temperature increase (shown by mean (line) and standard error of the mean (shade) of three repetitions) due to exposure to 50s continuous-wave RF energy exposure reveals that the presence of the copper mesh does not decrease (instead slightly increases) the induced RF electric field inside the brain.

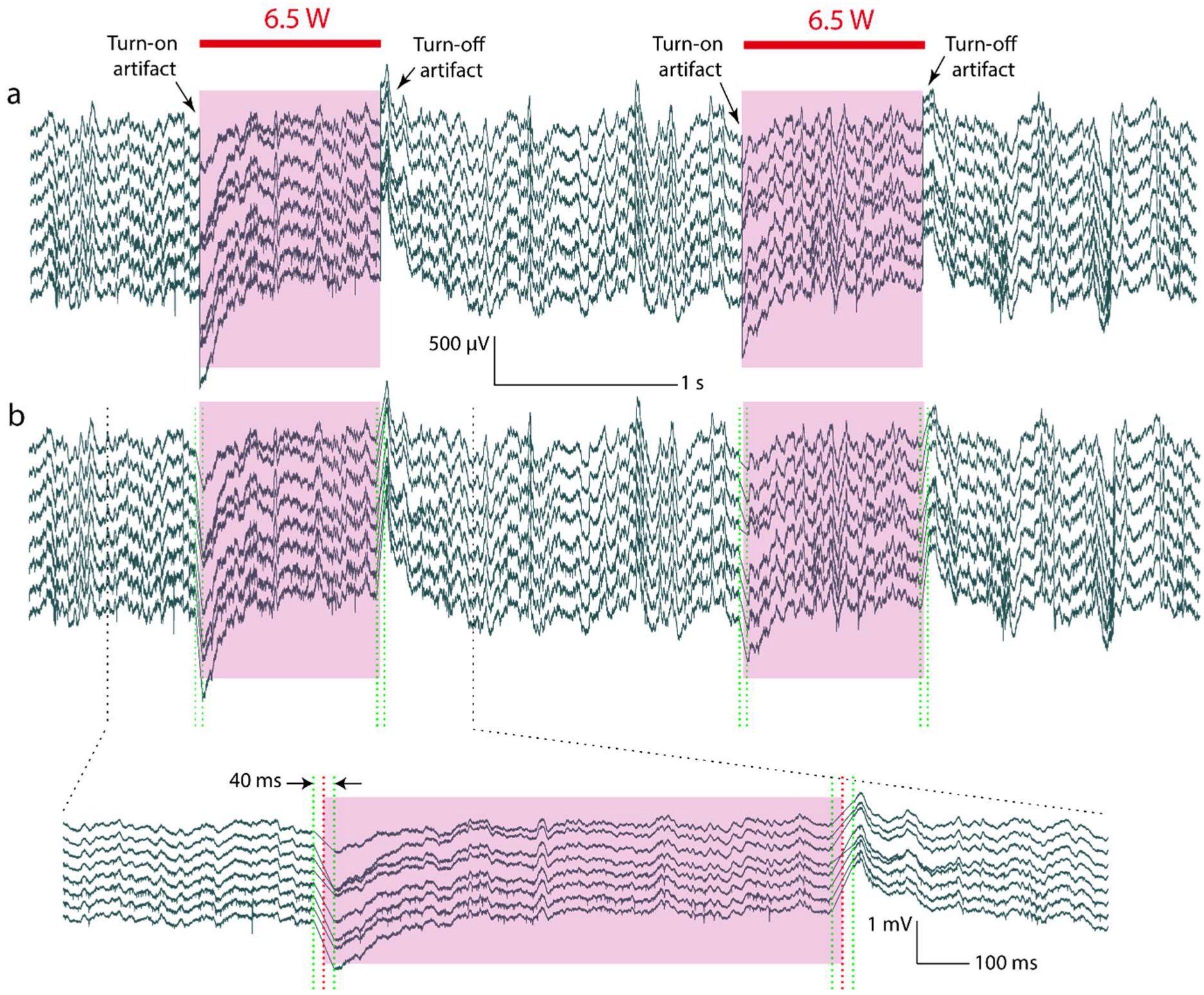

Supplementary Figure 2. **Removing RF-induced artifacts in electrophysiological recordings. a)** Example of recorded Local Field Potential (LFP) traces in mice hippocampus with RF induced artifacts consisting of sudden changes (at the RF turn-ON and turn-OFF moments) in the recorded potential followed by a decay period. Red bar indicates the RF stimulation period. **b)** As sudden changes in the LFP signal can affect the automatic detection of neural spikes, 20ms before to 20ms after the RF stimulation onset and offset points are replaced by a linear interpolation. Red bars and shadow rectangles indicate RF stimulation periods.

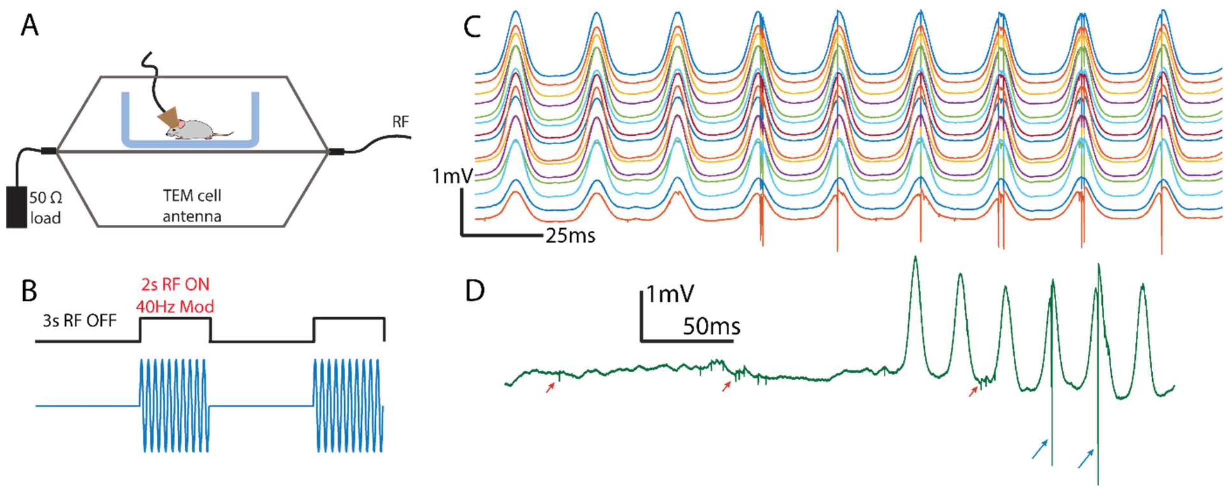

Supplementary Figure 3. **RF-induced population spikes in mice hippocampus. a)** Mice brain activity is recorded using electrophysiology while the animal, in its home cages, is placed inside a TEM cell antenna. **b)** Intermittent amplitude modulated (40Hz) RF is applied. **c)** Example of LFP traces during the RF exposure. Different colors show recording from different channels. Note the RF induced sinusoidal patterns on the LFP traces and the high amplitude population spikes. **d)** An example trace comparing population spikes (blue arrows) to single neuron spikes (red arrows).

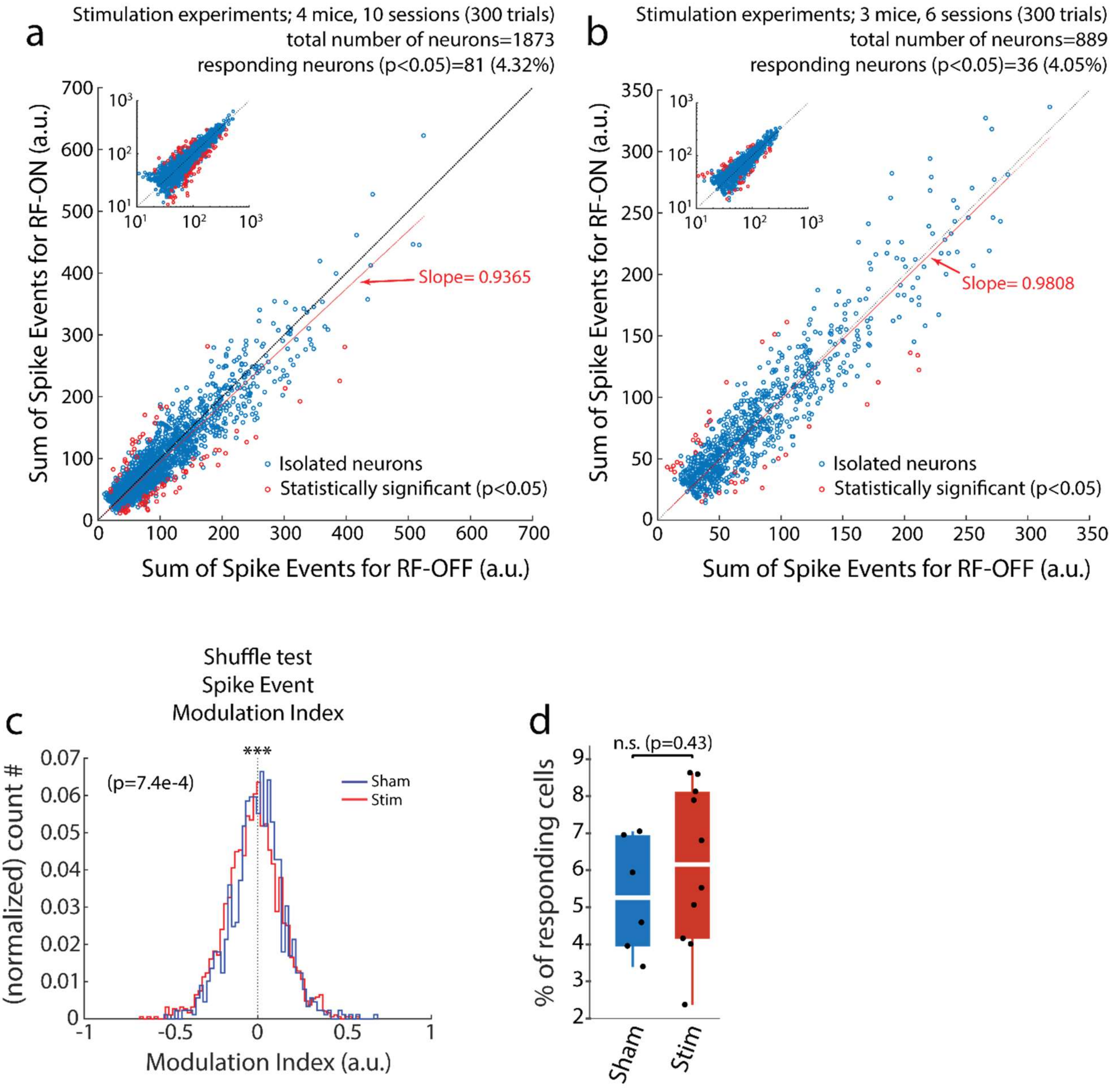

Supplementary Figure 4. **1-photon Ca^{2+} imaging in mice using the original UCLA Miniscope (V3). a)** RF response of all recorded clean (as per frequency spectrogram criteria in presented in Fig. 3b) neurons (ten 30-minute long sessions with 3s RF-ON 3s RF-OFF trials in 4 head-fixed mice with patch antenna (see Fig. 1d), 37.5 W at 950MHz). Activity of 81 out of 1873 neurons (4.32%) show statistically significant effect (shuffle test, $p<0.05$) during RF stimulation (red circles). The overall trend in all cells shows lower detected events during RF-ON versus RF-OFF periods. Inset shows the same plot in logarithmic scale. **b)** Same as in a for sham sessions: in six 30-minute long sessions with 3s RF-ON 3s RF-OFF trials in 3 head-fixed mice with non-connected patch antenna (37.5 W at 950MHz is fed in to a secondary antenna >2 m away). Activity of 36 out of 889 neurons (4.05%) show statistically significant difference (shuffle test, $p<0.05$; red circles). Inset shows the same plot in logarithmic scale. **c)** Comparison of distribution of modulation index (normalized histogram), calculated using number of occurrences of deconvolved spikes inferred from Ca^{2+} activity, between Sham and RF stimulation in all experiments (1873 neurons from Stimulation and 889 neurons from Sham; 30 minutes of 3s RF-ON, 3s RF-OFF trials; 6 Sham and 10 RF stimulation sessions in 3 and 4 mice, respectively). **d)** Comparison of percentage of RF responding cells (per session) with statistically significant ($p<0.05$) effect between Sham and RF stimulation in same data as in c. Note that

while these results are in apparent agreement with our electrophysiological results, the presence of the background light artifacts renders their interpretation questionable. Note: For the analysis of these experiments, we scrapped artifactual neurons with apparent peak at 0.16Hz in their frequency spectrum (as illustrated in Suppl. Fig. 5b). Further, we also discarded all analysis related to the area-under-the-curve as well as the height of the deconvolved spikes because of the potential effect of background light changes. We only compared events of deconvolved spikes neglecting their magnitude. Although the number of neurons significantly affected by the RF stimulation was comparable between stimulated (4.32% in 1873 cells in 10 sessions in 4 mice) and Sham (4.05% in 889 cells in 6 sessions in 3 mice) paradigms, we found a difference in the overall activity behavior (illustrated by the linear regression lines in Suppl. Fig. 4a and 4b). Also the distribution of the resulting modulation index across all cells were significantly different for Sham and Stimulation with latter being slightly shifted towards negative values (Suppl. Fig. 4c). These results suggested that RF stimulation accommodate suppression of activity in agreement with our electrophysiological results. However, the presence of the background light artifacts renders this interpretation nonviable.

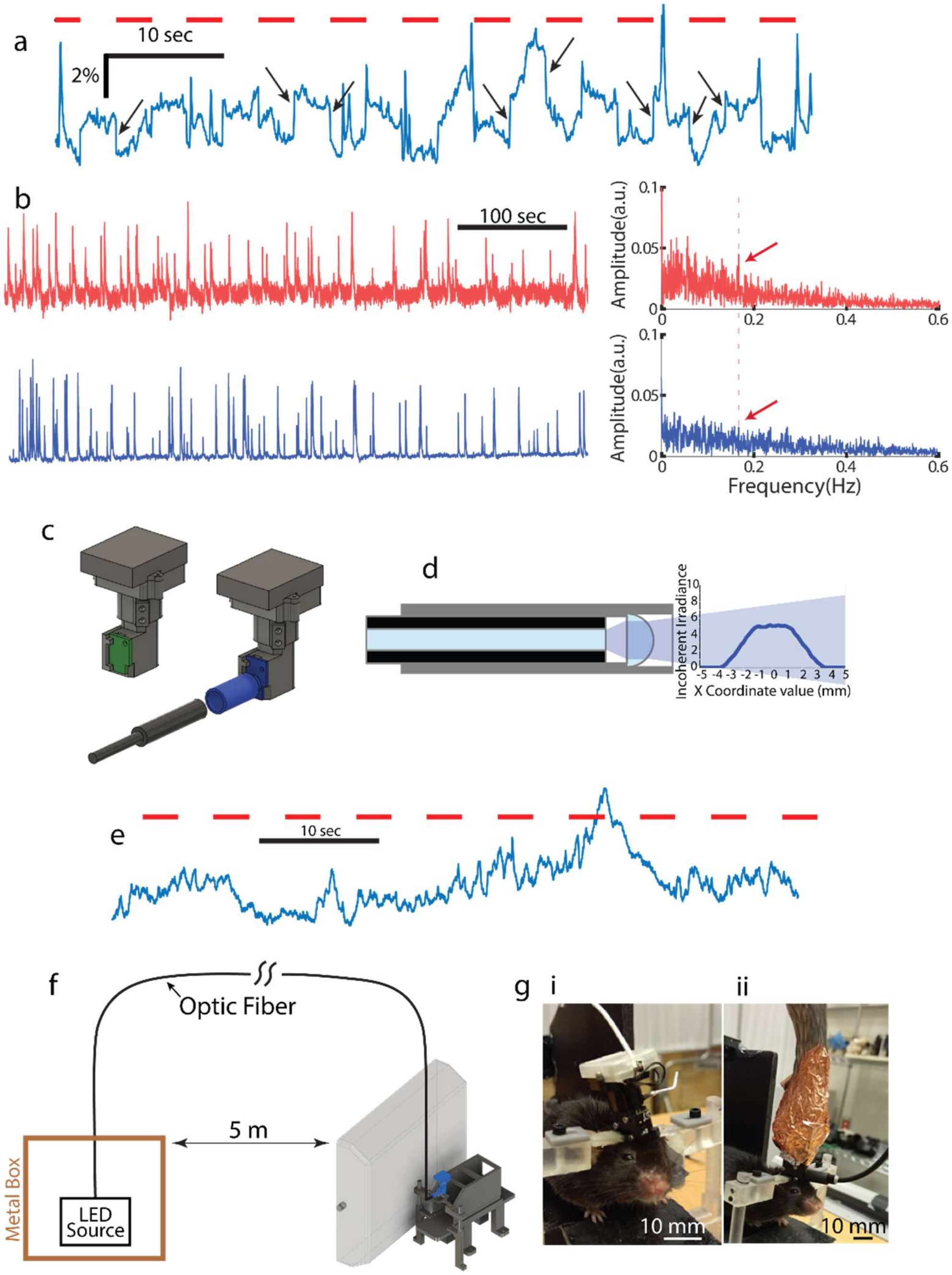

Supplementary Figure 5. **Fiber-coupled Miniscope for RF-artifact-free 1-photon imaging of neural activity. a)** Variation of sum of all pixels (entire imaged field) in an example recording session of hippocampal CA1 neurons, in response to 38W 3sON-3sOFF RF exposure (red bars indicate RF-ON epochs), using the original design of UCLA Miniscope. Note decrease of background florescence in presence of RF exposure but also the corresponding sudden changes in the accumulated trace (black arrows). Note that the changes in the background florescence in this example is around 2% of the ongoing fluorescence level. For most sessions the change is not visible in the recorded video. **b)** Example traces of Ca^{2+} transients (left) of two isolated neurons and their frequency spectrum (right). Top: Ca^{2+} activity trace of an example neuron which is affected by the RF-induced background florescence artifact (detected by presence of a peak at 0.16Hz [1/(6s)]); Bottom: an example cell with clean Ca^{2+} activity trace. In our first round of experiments, we discarded “noisy” cells and analyzed the effect of RF exposure on the activity of “clean” cells (see Suppl. Fig. 4). **c)** 3D schematic of the original UCLA Miniscope-v3 (left; its LED PCB is

highlighted in green) and the modified fiber-coupled version (right; a 3D-printed part, blue, for accommodating the added optic fiber). **d)** The custom-made fiber modification produces uniform light profile at the output. **e)** Same as A but using the modified fiber-coupled Miniscope. Note the absence of change in the background florescence. **f)** Schematic of the recording set-up using a fiber-couple LED source, shielded and placed >5 m from the recoding site. **g)** Picture of a head-fixed mouse for 1-photon imaging using the original UCLA Miniscope-v3 (i) and using the modified fiber-coupled version (ii).

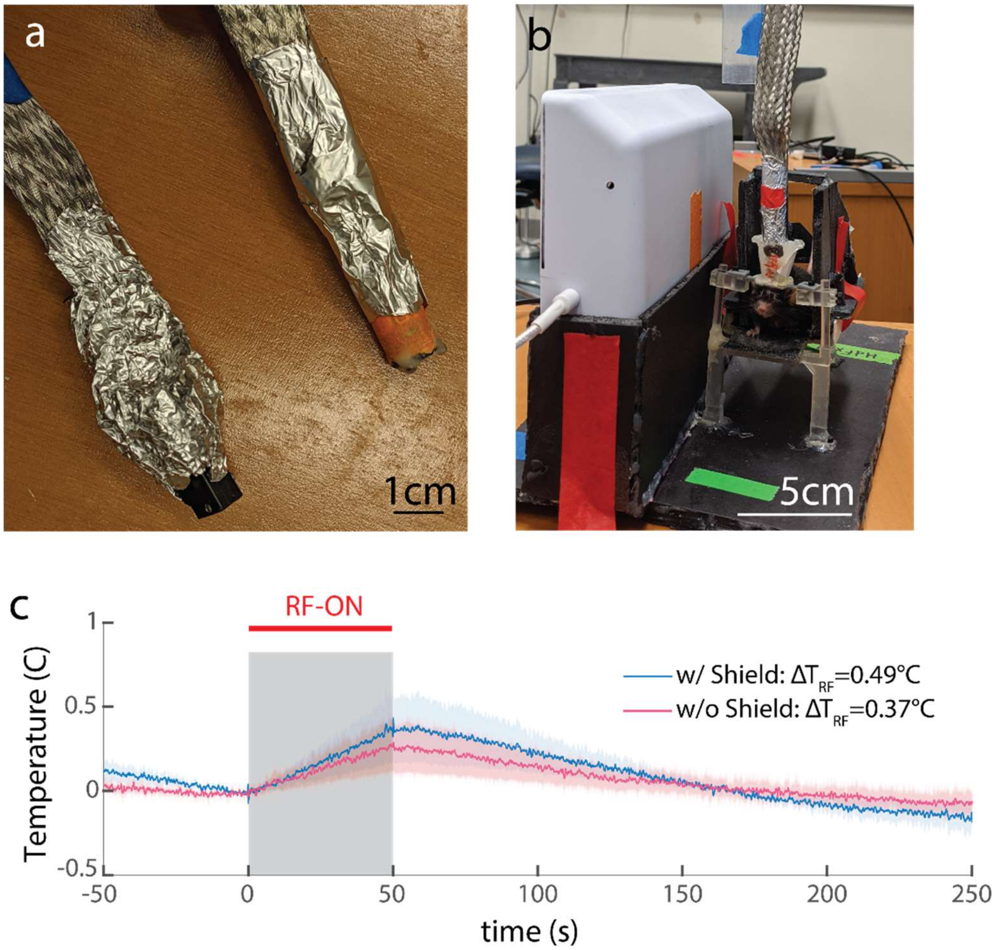

Supplementary Figure 6. **Adding shielding materials does not remove/reduce induced RF electric fields in the brain. a)** Illustration of actual shielding applied to the Miniscope body for 1-photon imaging experiments (left) and the shielding used for temperature measurements (right). **b)** Head-fixed animal preparation (with added shielding materials) for temperature measurement. **c)** RF-induced temperature rises (shown by mean (line) and standard error of the mean (shade) of three repetitions) in the mouse brain with (blue) and without (red) shielding materials. As the higher temperature rise indicates the in-situ RF electric fields are not only reduced by the shielding but are slightly enhanced.

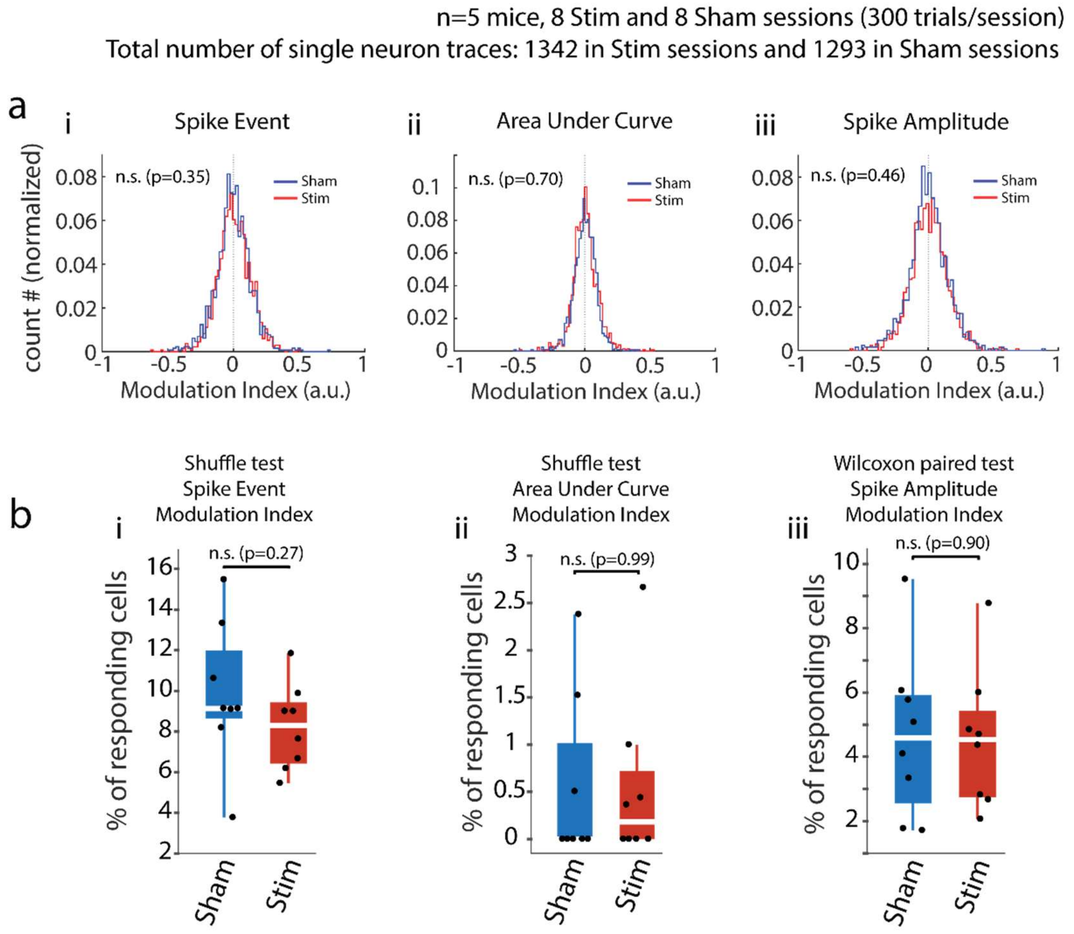

Supplementary Figure 7. **RF exposure does not affect ongoing neural activities of single neurons; Comparison of Single Sessions (related to Figure. 3). a)** Comparison of modulation index between Sham and RF stimulation (30 minutes of 3s RF-ON, 3s RF-OFF trials; 8 Sham and 8 RF stimulation sessions in 5 mice) calculated from different measured parameters: i) incidence of deconvolved spikes inferred from Ca^{2+} activity (neglecting the amplitude), ii) amplitude of Ca^{2+} activity trace (area under the curve) and iii) amplitude of deconvolved spikes (resulting plot is the same as Fig. 3h). **b)** Comparison of percentage of RF responding cells with statistically significant ($p<0.05$) effect between Sham and RF stimulation (30 minutes of 3s RF-ON, 3s RF-OFF trials; 8 Sham and 8 RF stimulation sessions in 5 mice). Group differences are examined by 3 different tests (plus 1 illustrated in Fig. 4h-j: shuffle test based on the amplitude of deconvolved spikes inferred from Ca^{2+} activity): i) shuffle test on the incidence of deconvolved spikes (neglecting the amplitude) ii) shuffle test on amplitude of Ca^{2+} activity trace (area under the curve), and iii) Wilcoxon sign-rank test on the amplitude of deconvolved spikes inferred from Ca^{2+} activity.

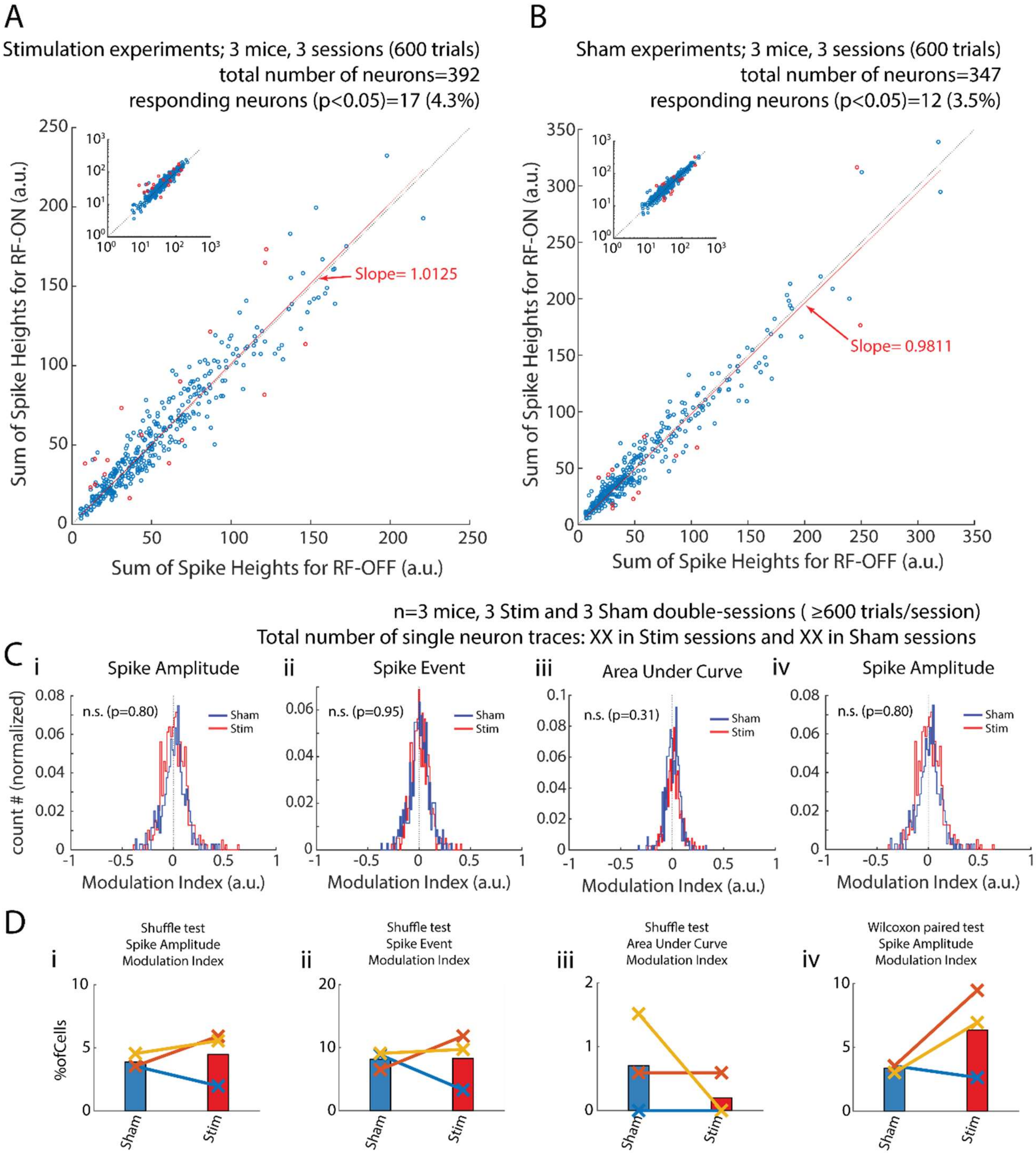

Supplementary Figure 8. **RF exposure does not affect ongoing neural activities of single neurons; Comparison of Double Sessions. a)** RF response of all recorded neurons (three 60-minute long concatenated double-sessions with 3s RF-ON 3s RF-OFF trials in 3 head-fixed mice with patch antenna (see Fig. 1d), 37.5 W at 950MHz). Activity of 17 out of 392 neurons (4.3%) show statistically significant effect (shuffle test, $p<0.05$) during RF stimulation (red circles). Inset shows the same plot in logarithmic scale. **b)** The fraction of statistically significantly affected neurons was similar in sham stimulation sessions: in three 30-minute long concatenated double-sessions with 3s RF-ON 3s RF-OFF trials in 3 head-fixed mice with non-connected patch antenna (37.5 W at 950MHz is fed in to a secondary antenna >2 m away). Activity of 12 out of 347 neurons (3.5%) show statistically significant difference (shuffle test, $p<0.05$; red

circles). Inset shows the same plot in logarithmic scale. **c)** Comparison of distribution of modulation index between Sham and RF stimulation in concatenated sessions when data from sessions recorded in consecutive days (> 600 trials of intermittent RF (3s ON, 3s OFF) were available (n = 3 mice). Group differences are examined by 4 different tests: i) shuffle test based on the amplitude of deconvolved spikes inferred from Ca^{2+} activity, ii) shuffle test on the incidence of deconvolved spikes (neglecting the amplitude), iii) shuffle test on amplitude of Ca^{2+} activity trace (area under the curve), and iv) Wilcoxon sign-rank test on the amplitude of deconvolved spikes inferred from Ca^{2+} activity. Colors refer to data from individual animals. **d)** Comparison of percentage of responding cells with statistically significant responses ($p<0.05$) between Sham and Stimulation (same data and for same tests as in c).

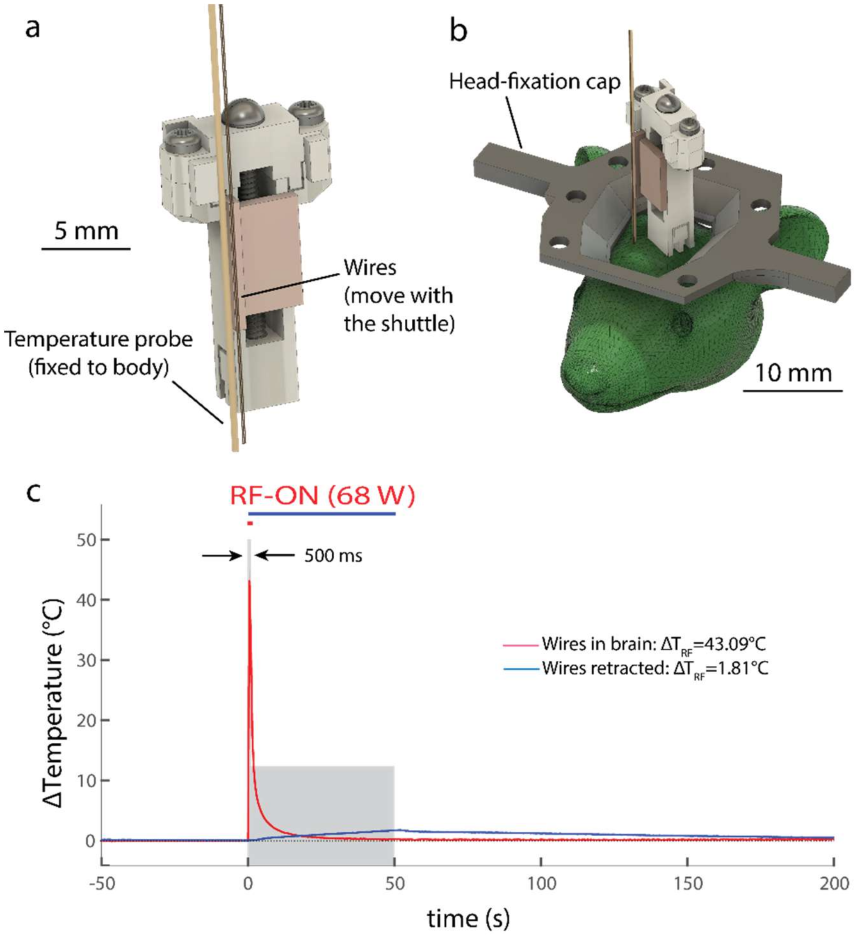

Supplementary Figure 9. **Metal in the brain induces a sharp and large local temperature increase. a)** 3D schematic of the implant preparation. A triplet of 50µm diameter tungsten wires is attached to the movable shuttle of a Microdrive. A small foot-print optical temperature sensor (300µm diameter, Osensa, Canada) is fixed to the stationary wall of the Microdrive. **b)** Illustration of the implanted temperature probe and wires. **c)** Temperature increase (shown by mean (line) and standard error of the mean (shade) of three repetitions) in head-fixed mouse brain due to exposure to continuous wave 68W RF energy (similar set-up as in Fig. 1d) for the metal in the brain (both temperature probe and wires are implanted) configuration (red) and after retracting the wires above the skull (blue). Note that in the-metal-in-the-brain configuration RF pulse is only 500 ms long (red bar), while in after retracting the metal wires from the brain, RF was continuously on for 50s (blue bar). Note the very large impact of the metal in the brain on temperature. Note also that 68W is several-fold higher than the power levels applied in our electrophysiological experiments (6.5 W).

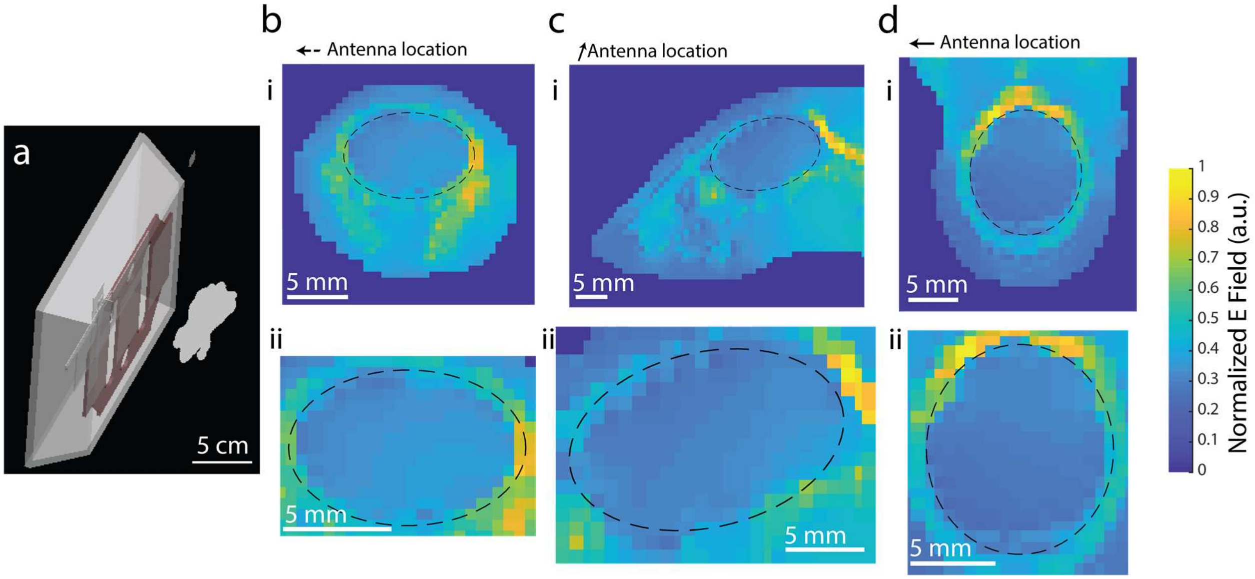

Supplementary Figure 10. **Numerical simulations to assess SAR and electric fields in mouse brain.** **a)** 3D schematic of the simulation setup in xFDTD software (see Methods) consisting of replication of the patch antenna (including conductive elements and outer plastic casing) and Digimouse mouse model (see Methods), arranged in a similar configuration as in Fig. 1d. Simulations are performed at 950 MHz (as in our experiments) for which the following values were used for brain tissue: relative permittivity of 49.9 and electrical conductivity of 1.12 (S/m). **b-d)** Normalized RF electric field distribution in the head of the mouse model on three 2D orthogonal planes (coronal (c), sagittal (d), and transverse (e)) at the coordinates related to the 1-photon imaging implant location: whole head images (i) and zoom views of the brain (ii). The same color bar on the right applies to all images.
