# Supplementary material for: Neuronal activity under transcranial radio-frequency stimulation in metal-free rodent brains in-vivo
Source: Commun Eng. 2022 Jul 1;1:15. doi: 10.1038/s44172-022-00014-7 (PMC10732550; doi:10.1038/s44172-022-00014-7)
Supplement: Supplementary file 1 [file 44172_2022_14_MOESM2_ESM.pdf]

nature portfolio

Corresponding author(s): Gyorgy Buzsaki, Omid Yaghmazadeh

Last updated by author(s): Apr 9, 2022

Reporting Summary

Nature Portfolio wishes to improve the reproducibility of the work that we publish. This form provides structure for consistency and transparency in reporting. For further information on Nature Portfolio policies, see our Editorial Policies and the Editorial Policy Checklist.

Statistics

For all statistical analyses, confirm that the following items are present in the figure legend, table legend, main text, or Methods section.

n/a	Confirmed	
☐	☒	The exact sample size (n) for each experimental group/condition, given as a discrete number and unit of measurement
☒	☐	A statement on whether measurements were taken from distinct samples or whether the same sample was measured repeatedly
☐	☒	The statistical test(s) used AND whether they are one- or two-sided<br>*Only common tests should be described solely by name; describe more complex techniques in the Methods section.*
☒	☐	A description of all covariates tested
☒	☐	A description of any assumptions or corrections, such as tests of normality and adjustment for multiple comparisons
☐	☒	A full description of the statistical parameters including central tendency (e.g. means) or other basic estimates (e.g. regression coefficient) AND variation (e.g. standard deviation) or associated estimates of uncertainty (e.g. confidence intervals)
☐	☒	For null hypothesis testing, the test statistic (e.g. F, t, r) with confidence intervals, effect sizes, degrees of freedom and P value noted<br>*Give P values as exact values whenever suitable.*
☒	☐	For Bayesian analysis, information on the choice of priors and Markov chain Monte Carlo settings
☒	☐	For hierarchical and complex designs, identification of the appropriate level for tests and full reporting of outcomes
☒	☐	Estimates of effect sizes (e.g. Cohen's d, Pearson's r), indicating how they were calculated

Our web collection on statistics for biologists contains articles on many of the points above.

Software and code

Policy information about availability of computer code

Data collection	Electrophysiological recordings were collected with the RHD2000 USB Interface Board from Intan Technologies (http://intantech.com/downloads.html?tabSelect=Software) and Neuroscope (http://neurosuite.sourceforge.net/).<br>1-Photon imaging data is collected by UCLA Miniscope-v3 and its specific data acquisition board and software (www.miniscope.org).<br>Temperature measurements data from Opsens probes were collected using a data acquisition system (RHD2000 USB Interface Board from Intan Technologies (http://intantech.com/downloads.html?tabSelect=Software)) connected to the specific measurement device (Accusens, Opsens).<br>Temperature measurements data from Osensa probes were collected using a specific software (OsensaView, Osensa, https://www.osensa.com/products).<br>Simulations were performed using a commercial 3D electromagnetic simulation software (xFDTD, Remcom, https://www.remcom.com/xfdtd-3d-em-simulation-software)
Data analysis	MATLAB script packages used in the analysis of this study can be downloaded from<br>https://github.com/MouseLand/Kilosort, and<br>https://github.com/etterguillaume/MiniscopeAnalysis, and custom scripts specific to this paper<br>can be found at https://github.com/omidyaghmazadeh/papers.

For manuscripts utilizing custom algorithms or software that are central to the research but not yet described in published literature, software must be made available to editors and reviewers. We strongly encourage code deposition in a community repository (e.g. GitHub). See the Nature Portfolio guidelines for submitting code & software for further information.

Data

Policy information about availability of data

All manuscripts must include a data availability statement. This statement should provide the following information, where applicable:

- Accession codes, unique identifiers, or web links for publicly available datasets
- A description of any restrictions on data availability
- For clinical datasets or third party data, please ensure that the statement adheres to our policy

The data that support the main findings of this study will be publicly available in the Buzsaki Lab Databank at https://buzsakilab.nyumc.org/datasets/YaghmazadehO/.

Field-specific reporting

Please select the one below that is the best fit for your research. If you are not sure, read the appropriate sections before making your selection.

☒ Life sciences ☐ Behavioural & social sciences ☐ Ecological, evolutionary & environmental sciences

For a reference copy of the document with all sections, see nature.com/documents/nr-reporting-summary-flat.pdf

Life sciences study design

All studies must disclose on these points even when the disclosure is negative.

Sample size	No statistical method was used to predetermined sample sizes but our sample sizes are similar to those typically reported in the field.
Data exclusions	No data was systematically excluded.
Replication	N/A
Randomization	N/A
Blinding	Blinding was not used in the present study.

Reporting for specific materials, systems and methods

We require information from authors about some types of materials, experimental systems and methods used in many studies. Here, indicate whether each material, system or method listed is relevant to your study. If you are not sure if a list item applies to your research, read the appropriate section before selecting a response.

Materials & experimental systems

n/a	Involved in the study	
☒	☐	Antibodies
☒	☐	Eukaryotic cell lines
☒	☐	Palaeontology and archaeology
☐	☒	Animals and other organisms
☒	☐	Human research participants
☒	☐	Clinical data
☒	☐	Dual use research of concern

Methods

n/a	Involved in the study	
☒	☐	ChIP-seq
☒	☐	Flow cytometry
☒	☐	MRI-based neuroimaging

Animals and other organisms

Policy information about studies involving animals; ARRIVE guidelines recommended for reporting animal research

Laboratory animals	For electrophysiology experiments: n=3 male mice (C57BL/6JxFVB, Charles River Lab), n=6 male rats (LongEvans, Charles River Lab).<br>For 1-photon imaging experiments: n=5 male mice (C57BL/6JxFVB, Charles River Lab)<br>For all temperature measurements: n=10 male mice (C57BL/6JxFVB, Charles River Lab), n=4 male rats (LongEvans, Charles River Lab).
Wild animals	N/A
Field-collected samples	N/A
Ethics oversight	IACUC NYU Medical Center

Note that full information on the approval of the study protocol must also be provided in the manuscript.
